# Physiotherapists’ experiences of osteoarthritis guidelines in primary health care – an interview study

**DOI:** 10.1186/s12875-021-01611-9

**Published:** 2021-12-30

**Authors:** Karin Sturesdotter Åkesson, Anne Sundén, Eva Ekvall Hansson, Kjerstin Stigmar

**Affiliations:** 1 Department of Health Sciences, Lund University, Lund, Sweden; 2 Department of Research and Education, Skåne University Hospital, Lund, Sweden

**Keywords:** Primary health care, Osteoarthritis, Physiotherapist, Patient education, Guidelines, Implementation, Interview study

## Abstract

**Background:**

Osteoarthritis is a common joint disease, globally. Guidelines recommend information, exercise and, if needed, weight reduction as core treatment. There is a gap between evidence-based recommended care for osteoarthritis and clinical practice. To increase compliance to guidelines, implementation was conducted. The aim of the study was to explore physiotherapists’ experiences of osteoarthritis guidelines and their experiences of implementation of the guidelines in primary health care in a region in southern Sweden.

**Methods:**

Eighteen individual, semi-structured interviews with physiotherapists in primary health care were analysed with inductive qualitative content analysis.

**Results:**

The analysis resulted in two categories and four subcategories. The physiotherapists were confident in their role as primary assessors for patients with osteoarthritis and the guidelines were aligned with their professional beliefs. The Supported Osteoarthritis Self-Management Programme, that is part of the guidelines, was found to be efficient for the patients. Even though the physiotherapists followed the guidelines they saw room for improvement since all patients with hip and/or knee osteoarthritis did not receive treatment according to the guidelines. Furthermore, the physiotherapists emphasised the need for management’s support and that guidelines should be easy to follow.

**Conclusion:**

The physiotherapists believed in the guidelines and were confident in providing first line treatment to patients with osteoarthritis. However, information about the guidelines probably needs to be repeated to all health care providers and management. Data from a national quality register on osteoarthritis could be used to a greater extent in daily clinical work in primary health care to improve quality of care for patients with osteoarthritis.

**Supplementary Information:**

The online version contains supplementary material available at 10.1186/s12875-021-01611-9.

## Background

Osteoarthritis (OA) in the hip and knee is one of the main causes of disability, globally [1]. The disease causes pain, stiffness, loss of function [1] and decreased quality of life [2]. Prevalence increases with age [3, 4] and OA affects about 10% of men and 18% of women over the age of 60 [4, 5]. However, many patients with OA in the hip and/or knee are of working age [6]. With extended life expectancy, prevalence increases which leads to an increased burden of the disease for both the affected individual [7], society and health care [1]. In Sweden, estimations show that in 2032, 30% of adults over 45 years are expected to have OA [8].

The evidence-based international non-pharmacological guidelines for treatment of OA include information, exercise and weight loss [9, 10]. The Swedish national guidelines are in accordance with the international guidelines [11, 12]. The guidelines highlight early diagnosis based on clinical examination and x-ray is only needed in certain circumstances such as if first line treatment has not been successful [13, 14]. Swedish physiotherapists (PTs) are often primary assessors for patients with musculoskeletal disorders such as OA [15]. This means that patients do not need a referral from a medical doctor (M.D.) to consult at PT [15] and that PTs are qualified to diagnose OA.

In Sweden, patients with hip and/or knee OA are offered the opportunity to participate in a Supported OsteoArthritis Self-Management Programme (SOASP), which is generally led by a PT in primary health care (PHC) [16]. The SOASP includes information and exercise and has been described elsewhere [16] and is evaluated through a national quality register called Better Management of Patients with OA (BOA) [16]. National quality registers contain data about patients concerning medical interventions, treatment and outcomes [17]. The registers provide an opportunity to monitor, enable learning, improvement and research to ensure quality of the Swedish health care system [17] and a vision is that the use of register data is integrated into daily clinical work [17]. The intention of the BOA register is that all patients with OA should be offered treatment according to current guidelines [18]. The BOA register contains patient reported outcome measures which enables systematic evaluation of the treatment for patients with OA and offers an opportunity for improvements of quality of care provided by PTs [18].

Systematic reviews have shown that there is a divergence between evidence-based recommended care for OA and clinical practice [19, 20] and less than 40% of patients with OA are offered the recommended first line treatment, i.e., education and exercise [21]. In 2013, it was estimated that about 8.7% of patients 45 years and older seeking health care due to OA in Sweden also participated in a SOASP [13]. A corresponding proportion for a region in southern Sweden, was almost the same (8.5%) [13], which means that only a small number of patients with OA in the hip and/or knee were offered treatment in accordance with the guidelines.

In order for health care to meet future needs, regional guidelines were produced in a region in southern Sweden, based on the international and national guidelines, that also include a recommendation to report to the BOA register [22]. According to the regional guidelines, PHC is primarily responsible for investigating, diagnosing, providing core treatment and additional treatment for patients with OA of the hip and/or knee [22].

The regional guidelines were implemented in PHC in a region in southern Sweden between the years 2016 and 2019 with the intention to increase health care providers’ compliance with the guidelines, and furthermore, to make treatment more accessible for patients and increase the reporting rate to the BOA register [22]. At the time of the implementation, there were about 150 PHC centres (tax-financed, public and private) in this region. All these PHC centres were given written information about the guidelines and about the implementation. Two coordinators with many years of clinical experience as PTs, of the SOASP and of reporting to the BOA register visited PHC centres to inform about the guidelines at workplace meetings where all categories of health care providers were present. The coordinators visited about 60% of the PHC centres at such workplace meetings between the years 2016 and 2019. Moreover, the coordinators offered the PTs individual support if needed and an education in how to organise a SOASP was provided for PTs on several occasions. In addition, an OA network for PTs responsible for offering the SOASP at their PHC centre was established to stimulate knowledge exchange, share experiences and news about OA treatment and from the BOA register. The OA network met twice a year during the implementation.

Implementation of new work procedures often takes several years [23]. Facilitating factors are, for example, involving personnel early in the process and also receiving support from the management [24]. Studies show that changes in working procedures are possible when implementation strategies are well planned and well performed [25]. Using an implementation theory or framework when planning, realising and evaluating implementation is recommended [26–28]. It is of great importance to evaluate an implementation in order to identify barriers and areas for improvement to increase knowledge for future implementation strategies [29–33]. Therefore, the aim of the study was to explore PTs’ experiences of the regional guidelines for treatment of OA of the hip and/or knee and their experiences of the implementation of the guidelines in a region in southern Sweden.

## Methods

An interview study was performed, analysed with qualitative content analysis (QCA).

### Participants and recruitment

A purposive sampling was used. Forty-three PHC centres a region in southern Sweden that had been visited by a coordinator between 2017 and 2018 during the implementation were contacted by email. The heads of departments (HOD) were informed about the study and were asked for permission to contact the PT responsible for the SOASP at the PHC centre to participate in the study. In total, 20 HODs did not reply despite a reminder being sent. One PHC centre had no PT at the time for the study.

The HODs at 22 PHC centres gave their consent and an email was sent with written information about the study to the PT responsible for the SOASP at these PHC centres. Approximately a week later, two co-authors, AS and KS who are both experienced registered PTs and PhDs trained in qualitative research, contacted the PTs by email to set a date for the interview. All participating PTs were given written and verbal information about the study and gave their written informed consent to participate in the study. In total, 18 PTs were interviewed once. Four were not included in the study due to the PTs not answering (two), declining to participate (one) and being on parental leave (one).

The characteristics of the participating PTs are described in Table 1.

**Table 1:** Characteristics of the participants

Characteristics	(***n*** = 18)
Sex	
Male	5
Female	13
Work experience in PHC^a^, mean (SD)	10.5 (7.2)
Work experience at this PHC^a^ centre, mean (SD)	4.4 (2.6)
Work experience with SOASP^b^, mean (SD)	4.25 (2.5)
Type of PHC^a^ centre	
Public	16
Private	2
Education in providing SOASP^b^	13

^a^*PHC* Primary Health Care

^b^*SOASP* Supported OsteoArthritis Self-Management Programme

### Data collection

Data were collected between February and September 2019. Eighteen semi-structured, individual interviews were conducted by AS (*n* = 10) and KS (*n* = 8), either at the participant’s workplace (*n* = 17) or at the Health Sciences Centre (*n* = 1) in Lund, Sweden, according to the participant’s preferences and with no one else present. All interviews were conducted in Swedish. An interview guide (see Additional file 1) was prepared, with inspiration from the framework Promoting Action on Research Implementation in Health Services (PARIHS) [34–36]. The PARIHS suggests that implementation is most likely to be successful when evidence is viewed as aligning with professional and patient beliefs, health care context is receptive to implementation and mechanisms are in place to facilitate implementation [37, 38].

The participants were interviewed once and the interviews lasted between 22 and 60 min, with a mean duration of 40 min (SD 13.2). All interviews were recorded using a tape recorder and were transcribed verbatim by the first author (KSÅ). There were no field notes made during or after the interviews. The transcripts were checked against the audio files twice. After the transcription, the record of participants was separated from the transcribed interviews ensuring anonymisation throughout the process of analysis.

### Data analysis

The transcribed interviews were analysed using QCA, with an inductive approach as described by Graneheim and Lundman [39, 40]. The method was suitable to gain a deeper understanding of PTs experiences and since it enabled us to explore opinions, ask open questions and to be able to approach data with focus on different and similar experiences. The transcripts as a whole were considered as units of analysis.

All 18 interviews were first re-read several times by three authors (AS, KS and KSÅ) to obtain an overview and an overall sense of the data. The text was then divided into meaning units, identified as “words, sentences or paragraphs containing aspects related to each other through content and context” [39]. In order to provide coherence, three authors (AS, KS and KSÅ) analysed two of the interviews separately regarding meaning units and two authors (KS and KSÅ) analysed two additional interviews regarding meaning units. The separate analysis was followed by a discussion by three authors (AS, KS and KSÅ). The remaining 14 interviews were divided into meaning units by the first author (KSÅ). Then, the meaning units in all 18 interviews were condensed and labelled with codes that were sorted in subcategories based on similar manifest content by the first author (KSÅ). The sorting and labelling of subcategories was followed by a discussion by three authors (AS, KS and KSÅ) until consensus was reached which led to some subcategories being re-sorted and re-labelled. The analysis process went back and forth. Finally, after further abstraction, subcategories were sorted into categories reflecting their content. Three authors (AS, KS and KSÅ) were engaged in labelling the categories. All authors (AS, KS, EEH and KSÅ) were involved in discussing the results and the conclusion. The participants did not provide feedback on the findings. However, when closing each interview, the interviewers gave a summary of the interview, and the participants were able to modify. Examples of meaning units, condensed meaning units, codes, subcategories and categories are provided in Table 2.

**Table 2:** Examples of the analysis process with meaning unit, condensed meaning unit, code, subcategory and category

Meaning unit	Condensed meaning unit	Code	Subcategory	Category
Then it is the younger ones, those who are still more active and simply are unable to leave their place of work (ID 1)	It is the younger ones who are more active and unable to leave their work	Younger are unable to leave their place of work	The Supported OsteoArthritis Self-Management Programme does not suit all patients with OA	The Supported OsteoArthritis Self-Management Programme is overall a well-functioning part of the regional guidelines but there is room for improvement
I actually don’t really know what the nurses know about osteoarthritis. I mean when they meet patients and on the phone and what they might say to the patients (ID 2).	Don’t know what nurses know about osteoarthritis and what they say to patients	What do nurses know about osteoarthritis	Guidelines must be easy to follow	Management plays a key role when it comes to guideline compliance

The meaning units, codes, subcategories, categories, and quotes were translated into English by the first author (KSÅ). Thereafter, a professional English reviser was consulted who edited the manuscript which was also proofread by a bilingual reviser (English and Swedish). The language editing was done in cooperation with the first author (KSÅ) so that the English translation remained close to the Swedish text in the interviews since this is a focus in QCA.

This study was conducted by four female PTs and researchers: one professor (EEH), one associate professor (KS), one PhD (AS) and one PhD student (KSÅ). Two researchers have on-going clinical practice (EEH, KSÅ) and two are experienced in interviewing and doing qualitative studies (KS, AS). The study was reported in accordance with the checklist of Consolidated Criteria for Reporting Qualitative Research (COREQ) [41] (see Additional file 2). The software programme NVivo 12 was used in the analysis.

## Results

The analysis resulted in two categories and four subcategories.

### The Supported OsteoArthritis Self-Management Programme is overall a well-functioning part of the regional guidelines but there is room for improvement

The SOASP was already established as part of treatment for patients with hip and/or knee OA in PHC. The PTs experienced a great need for the SOASP and saw that this treatment was often requested by patients. In addition, the SOASP provided PTs with an effective work procedure for patients with hip and/or knee OA. However, parts of the work procedure could be improved.

#### Physiotherapists are confident in their professional role and believe in the guidelines

The PTs were confident in the role as primary assessor and in clinically diagnosing patients with hip and/or knee OA. They felt competent enough to know when a patient needed a referral to an M.D. or orthopaedist.

And then we have good competence to diagnose … .and to start them off with an efficient treatment (ID 3).

The guidelines were aligned with professional beliefs for treatment of OA and most patients’ believed in the treatment. The SOASP was a good support for the PTs in treating the patients and the material used in the programme was useful. It was an effective programme for the patients who learned how to cope successfully with their disease. Many patients experienced decreased pain and improved physical condition. Some patients declined surgery after participating in the SOASP. Moreover, having a group of patients with the same diagnosis made the group more close-knit and the patients learned from and supported each other. However, while the need for the SOASP was great and PTs wished they could offer the programme more frequently, time was limited.

I think it is an effective way to treat the patient and when they get the information offered through the SOASP I think they feel more confident and they do not have to seek health care as much afterwards … so for the time it takes to deliver the SOASP we do gain in some way. I believe that more of the patients felt better, they felt stronger after having exercised and had less pain and anxiety (ID 5).

#### The Supported OsteoArthritis Self-Management Programme does not suit all patients with osteoarthritis

Even though the PTs found the SOASP to be effective for most patients with hip and/or knee OA, they also saw that not all patients with OA participated. The SOASP seemed to be less relevant to younger patients of working age with OA who were reluctant to acknowledge to having the OA diagnosis. Moreover, younger patients also had difficulties in attending the SOASP during the daytime and had problems finding time to exercise.

Sometimes, in order to reach the younger patients, we have been able to use preventive sick leave, and that is no problem to get. Patients often do not want to leave their place of work, which is a barrier. They would rather work and they do not want this preventive sick leave. However, it has worked very well for those who have taken it and then we reach the younger patients as well (ID 8).

According to the PTs, the SOASP was not efficient for patients with severe pain and disability and when surgery was planned. Moreover, patients with native languages other than Swedish were considered more difficult to reach and the importance of enabling participation for patients who spoke a foreign language was highlighted. When needed, the PTs engaged interpreters to communicate with non-native Swedish patients participating in the SOASP. Physiotherapists with experience of including an interpreter in the SOASP thought that this worked well overall. However, not all these interpreters were authorised leading to an insecurity among PTs regarding the information delivered to the patients. Hence, for these reasons, written information in more languages than the existing ones would be appropriate and beneficial. In addition, the PTs thought it was challenging to meet patients with unrealistic expectations, such as getting rid of the pain completely, and patients with a lower educational level. Therefore, treatment was sometimes individualised according to patients’ needs.

And most of the patients in my SOASPs have only basic education and nothing more, and it might be even harder to absorb and learn if you are not used to going to school, or if you are not even able to write in your own language and then you are supposed to read in another language … .and looking at pictures and so on can also be difficult to understand (ID 7)

The PTs experienced other challenges as well: when patients were doubtful about the OA diagnosis, especially when it was not confirmed on an x-ray or when patients preferred to see an M.D. since more passive treatments, x-ray, medication and sick leave were requested. In addition, some patients wanted a fast recovery and did not want to participate in the SOASP. The PTs acknowledged that some patients needed further actions such as pain relief, before even being able to participate in the SOASP, or a longer period of supported training, which made it difficult to finish the treatment after the SOASP.

### Management plays a key role when it comes to guideline compliance

The importance of having the interest, support and engagement from the management at both PHC level and regional governance level was emphasised by the PTs in order for all personnel to work optimally according to guidelines. Furthermore, management support was crucial when it came to sustaining knowledge, evaluation and development.

#### More support is needed in order to prioritise and enable evaluation and development

According to the PTs, the HODs knew about the SOASP, although it was not prioritised. While the PTs did not see a need for explicit control, they would appreciate it if the HODs showed more interest in their work. Much of the HODs’ attention was on the PTs treating as many patients as possible, and therefore, reporting to the BOA register and evaluation was not prioritised. Reporting data to the register was time consuming for the PTs and development work was falling behind due to lack of time. The results from the register were not requested by the HODs and workplace meetings were mostly about the budget at the PHC, not quality of care. Only a few PTs that reported to the BOA register actually used the data for analysis, evaluation and development. It was not clear to the PTs that management at regional governance level was responsible for the implementation. However, PTs experienced enough support from the HODs to be able to offer the SOASP in accordance with the guidelines.

Because there are many patients seeking our care, it is sometimes difficult to allocate time to development work … since we need to take care of the patients in the clinic (ID 5).

#### Importance of knowledge and understanding of the guidelines

The PTs experienced that not all health care personnel followed the guidelines. Consequently, not all patients with hip and/or knee OA saw a PT and some patients only saw an M.D. It was unclear to the PTs what knowledge M.D.s and registered nurses (R.N.) had about OA. Therefore, the PTs saw a need for improved triage and this was discussed continuously. In addition, the PTs expressed that there were still M.D.s that send referrals to x-ray instead of to the PT, which delayed treatment according to the guidelines. Knowledge of the guidelines was vital; otherwise, it was impossible to follow them. Due to a high employee turnover in PHC, the PTs thought that information about the guidelines should be repeated continuously. Locum doctors did not follow the guidelines and triage was affected when there were new personnel. The electronic medical record was suggested to be used to support and facilitate compliance with the guidelines. Nevertheless, collaboration with colleagues and other professionals overall worked well.

It should be easy to follow the guidelines, but that is not the case today. It is quite difficult to follow the guidelines because then you have to have read them as well as remember them. Moreover, you have to remember them in a stressful situation too. Therefore, it is quite difficult to follow the guidelines today. Actually, a bit too difficult since you have to be very much updated and very interested to be able to fully follow the guidelines (ID 14).

The information about the guidelines given by the coordinator at the workplace meetings was considered useful and it was beneficial that it came from a person from outside the PHC centre. However, PTs thought that M.D.s listen more carefully to information from other M.D.s and since the coordinators were PTs, this might have limited the effect of the information given. The PTs profoundly expressed the need for equal yet individualised care, although they realised that implementation takes time. Moreover, it was important to be able to exchange knowledge with other PTs working with patients with OA and the SOASP. Therefore, the network meetings arranged within the implementation were much appreciated and gave valuable opportunities for exchanging knowledge and experience.

And then the lack of knowledge, I mean if you see a medical doctor who does not know what to do about OA … and sometimes medical doctors send a referral to the orthopaedist … and the referral is sent back since the patient has not seen us instead. Therefore, there is still a lack of knowledge about the treatment of osteoarthritis when it comes to the other professionals (ID 10).

## Discussion

In this interview study, we found that the SOASP was generally a well-established and functioning part of the regional guidelines and that the PTs saw a great need for the treatment. Moreover, the PTs were confident in their professional role and as a primary assessor for patients with OA in the hip and/or knee. However, the SOASP was not seen as appropriate for all patients with OA. Moreover, management support was considered important when it came to compliance with the guidelines, and to enable evaluation, development and sustaining knowledge among the health care personnel.

In our study, PTs described compliance with the guidelines on several points. The PTs expressed confidence in being the primary assessor and when clinically diagnosing patients with OA of the hip and knee. Our result is in line with another Swedish study where PTs in PHC centres self-reported to be confident in managing patients with OA regarding assessment, treatment and education [42]. However, international studies have shown that PTs wanted x-rays to support a diagnosis [43, 44]. Since an x-ray is not needed when diagnosing OA of the hip and knee according to international guidelines [9–11], it is encouraging that the PTs in our study felt confident in clinically diagnosing OA.

The SOASP was first introduced in Sweden in 2008 [45] and was generally already established in PHC. Furthermore, many PTs in Sweden have undertaken an education in order to offer the SOASP in accordance with the guidelines for the treatment of OA [46]. In addition to knowledge about OA, the education provides the PTs with digital material to be used when offering the SOASP to patients [46]. The majority of the participating PTs in our study had undertaken the education and this might be one reason why PTs clearly expressed confidence in assessing and diagnosing OA and to their compliance with the guidelines when it comes to offering the SOASP. One other study has shown that PTs in general show compliance with the guidelines for the treatment of OA in PHC when it comes to exercise and education [42]. This is not surprising since there is evidence that exercise is beneficial for patients with OA and exercise is included in the guidelines [11].

The PTs saw the SOASP as being efficient, useful, and beneficial for patients with OA in the hip and/or knee. Therefore, they might also be motivated to provide the treatment. Studies show that compliance with guidelines is positively affected by professional beliefs [37, 38, 47].

According to the PTs, the SOASP was not appropriate for all patients with OA. Patients of younger age, with native languages other than Swedish and with lower levels of education were more difficult to reach within the SOASP. Studies have highlighted the importance for all patients to understand information in order to be empowered and be active participants in their own health care [48, 49]. The PTs in our study emphasised the importance of providing equal care to all patients with OA but also the need to individualise treatment. Other studies have previously suggested a more person-centred approach in treatment [49–51]. Criticism of guidelines is, for example, that they are not patient-centred but disease focused [51, 52]. Furthermore, guidelines do not consider comorbidities which make them difficult to follow in clinical practice [51]. Our result might indicate that the SOASP could be further developed to be more individualised.

The support from management was found to be important both regarding compliance with the guidelines for all health care personnel and also regarding knowledge, evaluation and development. Such findings on management support are not surprising or new but it rather confirms previous research [32, 53–55]. Even so, it is important to study the ways in which management is important since this can differ between different contexts.

The PTs in our study experienced that there was often more attention on the production of care and budget at the PHC centre, rather than on the quality of care. This is in line with another Swedish study where physiotherapy managers experienced more focus on budgets and production of care than on evidence-based practice from higher management levels [56]. In our study, the PTs wished for more management support to be able to evaluate and develop the work process. The PTs continued to report in the BOA register even though it was time consuming, and HODs did not ask for results. Having time to analyse and use knowledge based on local data reported in the BOA register could be a useful way to ensure quality of care for patients with OA of the hip and knee and would be in line with the vision for how the national quality registers could be integrated and used in the daily clinical workflow [17].

According to the interviewed PTs, not all health care personnel followed the guidelines. Some patients were not referred to a PT, some came to the PT late in their disease development and after having an x-ray. This is in line with other studies showing that M.D.s were unaware of guidelines for OA [47] and overused x-ray in diagnosing OA [57]. Guidelines should be easy to follow [47] and in an attempt to facilitate compliance and make it easier for M.D.s, flowcharts have been created [58]. Since the PTs in our study already followed the guidelines, perhaps the implementation should have focused more on including other health care personnel, i.e., M.D.s and R.N.s and also HODs. This could possibly have a greater impact on providing evidence-based care for patients with OA in the long run.

### Strengths and weaknesses of the study

Several steps were taken to ensure trustworthiness [59]. A purposive sample was used i.e., PHC centres and participants were selected based on their experience of having had a coordinator at a workplace meeting informing all health care personnel about the regional guidelines. The sample of participants was geographically spread and included PTs with longer and shorter clinical work experience as PTs and experience of the SOASP. Quotes were presented in a way which prevented identification of the participants. Several researchers were involved in the analysis process and all authors were involved in discussing the results and the conclusion.

The first author, KSÅ, has experience from working as a PT in PHC and of working according to the guidelines. KSÅ was actively involved in the implementation as being one of the coordinators. However, the interviews were conducted by two co-authors that had not been involved in the implementation in order to reduce the risk of bias. Our intention with the study was to explore the PTs’ experiences of the regional guidelines for treatment of OA of the hip and/or knee and their experiences of the implementation of the guidelines with an inductive approach, not to evaluate an implementation process in relation to a specific framework. However, using the PARIHS framework as inspiration when preparing the interview guide ensured that relevant topics were illuminated during the interview, for example the PTs’ and patients’ beliefs about the guidelines and the PTs’ experiences of barriers and facilitators when working in accordance with the guidelines. Hence, our findings correspond to parts of the PARIHS framework as would be expected.

The description of the setting, recruitment, characteristics of the participants and process of analysis has been presented in detail both in the Method section and in Table 1 and Table 2 to enable evaluation of transferability as well as credibility.

We assessed that 20 participants would be sufficient to provide a variation in experiences and yet be a manageable amount of data. Eighteen interviews were conducted which we considered to be close to the desired sample.

A limitation with the study is that a pilot interview was not conducted. A pilot interview was planned but was cancelled. However, we believe this was compensated to some extent since potential changes in the interview guide were discussed by three of the authors after the first two interviews were conducted. No changes were made after these first interviews. Another limitation is the difference in the length of the interviews that might have affected the amount of data. Moreover, there were only two private PHC centres represented which somewhat reflects the fact that fewer private PHC centres invited the coordinators to their PHC centre during the implementation.

## Conclusion

The PTs believed in the guidelines and were confident in providing first line treatment to patients with OA. Still, there was room for improvement. The SOASP did not suit all patients and therefore an individualisation of treatment for patients with OA was sometimes needed. Moreover, compliance to guidelines could be increased since not all health care providers followed them. Hence, there is a need to repeat information about the guidelines to all health care providers and management. We believe that data from the national quality register, BOA, could probably be used to a greater extent in daily clinical work in PHC for continuous learning and evaluation and to improve quality of care for patients with OA.

## Supplementary Information

**Additional file 1.**

**Additional file 2.**

## Data Availability

The dataset generated and analysed during the current study is not publicly available due to confidentiality policies but are available from the corresponding author on reasonable request.

## Abbrevations

OA: Osteoarthritis
PT: Physiotherapist
M.D.: Medical doctor
SOASP: Supported OsteoArthritis Self-Management Programme
PHC: Primary Health Care
BOA: Better Management of patients with OsteoArthritis
QCA: Qualitative Content Analysis
HOD: Heads of Department
PARIHS: Promoting Action on Research Implementation in Health Services
COREQ: Consolidated Criteria for Reporting Qualitative Research
R.N.: Registered Nurse
