# Supplementary material for: Physiotherapists’ experiences of osteoarthritis guidelines in primary health care – an interview study
Source: BMC Fam Pract. 2021 Dec 30;22:259. doi: 10.1186/s12875-021-01611-9 (PMC8717645; doi:10.1186/s12875-021-01611-9)
Supplement: Supplementary file 1 — Additional file 1. [file 12875_2021_1611_MOESM1_ESM.docx]

**Interview guide**

**Introduction** **and background questions**

- Interviewer repeats information about study and consent
- How long have you been working as a physiotherapist? At this primary health care (PHC) centre? With the Supported OsteoArthritis Self-Management Programme (SOASP)?

**Main topics and probing questions**

*Regional guidelines for treatment of osteoarthritis (OA)*

- What are your beliefs regarding the regional guidelines for the treatment of OA? Are the guidelines aligned with your professional beliefs on the treatment of OA?
- What are the strengths and weaknesses of the guidelines?

*Experience of offering the SOASP*

- What is your experience of working with the SOASP according to the guidelines for the treatment of OA?
- What is your experience of evaluation by using data from the “Better Management of Patients with OA” register?
- What is your clinical experience regarding the treatment of OA?
- What is your experience of patient preferences regarding the treatment of OA? Are the guidelines aligned with patients´ beliefs on the treatment for OA?

*Experience of implementation strategies*

- What do you know about the implementation intervention?
- What is your experience of the implementation strategies used to support the implementation of the regional guidelines?

*Experience of barriers and facilitators at local PHC level, professional level and organisational level*

- How does the collaboration with other professionals work regarding patients with OA at your PHC centre?
- What is your experience of barriers and facilitators when working with the SOASP according to the regional guidelines (at local PHC level, professional level and organisational level)

**In conclusion**

- Summary of interview by the interviewer
- Is there anything you would like to add?
